# Beyond the numbers: Human attitudes and conflict with lions (*Panthera leo*) in and around Gambella National Park, Ethiopia

**DOI:** 10.1371/journal.pone.0204320

**Published:** 2018-09-25

**Authors:** Fikirte Gebresenbet, Hans Bauer, Jacqueline M. Vadjunec, Monica Papeş

**Affiliations:** 1 Department of Integrative Biology, Oklahoma State University, Stillwater, OK, United States of America; 2 Wildlife Conservation Research Unit, Department of Zoology, University of Oxford, United Kingdom; 3 Department of Geography, Oklahoma State University, Stillwater, OK, United States of America; 4 Department of Ecology and Evolutionary Biology, University of Tennessee, Knoxville, TN, United States of America; U.S. Geological Survey, UNITED STATES

## Abstract

Human-lion conflict is one of the leading threats to lion populations and while livestock loss is a source of conflict, the degree to which livestock depredation is tolerated by people varies between regions and across cultures. Knowledge of local attitudes towards lions and identification of drivers of human-lion conflict can help formulate mitigation measures aimed at promoting coexistence of humans with lions. We assessed locals’ attitudes towards lions in and around Gambella National Park and compared the findings with published data from Kafa Biosphere Reserve, both in western Ethiopia. We used household interviews to quantify livestock loss. We found that depredation was relatively low and that disease and theft were the top factors of livestock loss. Remarkably, however, tolerance of lions was lower around Gambella National Park than in Kafa Biosphere Reserve. Multivariate analysis revealed that education level, number of livestock per household, livestock loss due to depredation, and livestock loss due to theft were strong predictors of locals’ attitude towards lion population growth and conservation. We show that the amount of livestock depredation alone is not sufficient to understand human-lion conflicts and we highlight the importance of accounting for cultural differences in lion conservation. The low cultural value of lions in the Gambella region corroborate the findings of our study. In combination with growing human population and land-use change pressures, low cultural value poses serious challenges to long-term lion conservation in the Gambella region. We recommend using Arnstein’s ladder of participation in conservation education programs to move towards proactive involvement of locals in conservation.

## Introduction

Conserving large carnivores is a global challenge in the face of increasing human population and associated land-use and land-cover changes. Human-lion conflict, a situation in which people retaliate against lions that actually or presumably affect peoples’ livelihoods or well-being, is a major cause for the reduction of lion populations throughout Africa [1–3]. Conflicts escalate as the frequency of human-lion interactions increases with human population growth and encroachment into lion habitats, ultimately resulting in range contractions and decreasing numbers of lions [4]. Poor animal husbandry practices create further opportunities for livestock depredation as humans move into or close to protected areas in search of resources [2] and lions disperse to adjacent areas in search of prey [5].

Predators, such as lions, may represent actual or perceived threats to humans or livestock [6, 7]. Human attitudes, behaviors, and perceptions towards carnivores, resulting from complex social and cultural settings, are key to understanding human-carnivore conflicts [8]. Local perceptions towards carnivores can be shaped by various factors, including the amount of livestock loss due to depredation, level of wealth [8] and education [9], but certainly also culture [9, 10]. Peoples’ personal experience of livestock depredation has been linked to negative attitudes towards carnivores, resulting in intentional killings to reduce their numbers [3, 11]. The lack of lion related benefits for locals amplifies the notion that lions are conserved at the cost of locals’ safety and economic subsistence. For example, Romañach et al [12] reported that locals claimed they would be more tolerant of depredations if they benefited from carnivore conservation actions, and members of the community with income from tourism had positive attitudes towards predators.

People’s tolerance towards carnivores is influenced by attitudes and perceptions that are deeply rooted within cultures [13]. Positive engagement with local cultural contexts facilitates wildlife conservation [14]; Hazzah et al [15] for example have provided evidence that lion killing can be reduced by working within the cultural context of the Maasai. Participatory approaches that engage locals from planning to implementation of conservation actions promote legitimacy of proposed solutions to human-wildlife conflicts. However, if locals are passive participants, the participatory approach remains nominal and lacks power-sharing and partnership [16]. The classic carrot-and-stick approach, rewarding desired and disciplining undesired human behaviors, can be used in the mitigation of human-wildlife conflicts [17]. Community participation and collaboration can become effective if guided through the levels of participation proposed by Arnstein [18]. These levels range from a non-participation stage (in which local populations will only be educated on the importance of carnivores) to citizen power and empowerment (where individuals actively participate in the conservaion process and hold the managerial decision-making capabilities) [18].

The African lion (*Panthera leo*) is listed as “Vulnerable” by the International Union for Conservation of Nature (IUCN), with a 43% population decline between 1993 and 2014 [1, 19], and majority of lion populations exising in protected areas [20]. Several studies exist on various aspects of lion conservation in different African countries [21–25]. In Ethiopia, lions are generally considered important socially and culturally [26], however substantial gaps remain in our knowledge of Ethiopian lions. The need for deeper knowledge is critical because declines in Ethiopian lion populations [26] could have crucial effects on lion conservation, as southern Ethiopia is the only bridge connecting East and Central African lion populations [27].

In this study, we surveyed the attitudes of people towards lions in and around Gambella National Park (GNP), in Gambella Regional State (Gambella hereafter), western Ethiopia. We followed the definition of Ajzen and Fishbein [28] for attitude: an evaluation of an object by an individual. We compared our findings from in and around GNP with published data from Kafa Biosphere Reserve in southwestern Ethiopia [10]. Following the South Sudanese civil war that started in 2013, Gambella became home to approximately 400,000 refugees and asylum seekers from neighboring South Sudan [29]. This influx of people, combined with natural population growth and long term trends of immigration to Gambella from the Ethiopian highlands, could increase the pressure on GNP’s resources and affect lion populations directly but more importantly indirectly, through prey depletion. Gambella is considered to have a viable lion population in Ethiopia [26], but the level of human-lion conflict and its cultural and economic dimensions have not been studied. Understanding region- and time-specific attitudes and behaviors towards carnivores is needed to develop successful conservation measures [30]. We therefore surveyed the attitudes of people in and around GNP towards lions and compared our findings with those of a published study from Kafa Biosfere Reserve, southwestern Ethiopia [10].

## Methods

### Study area

**Fig 1:**
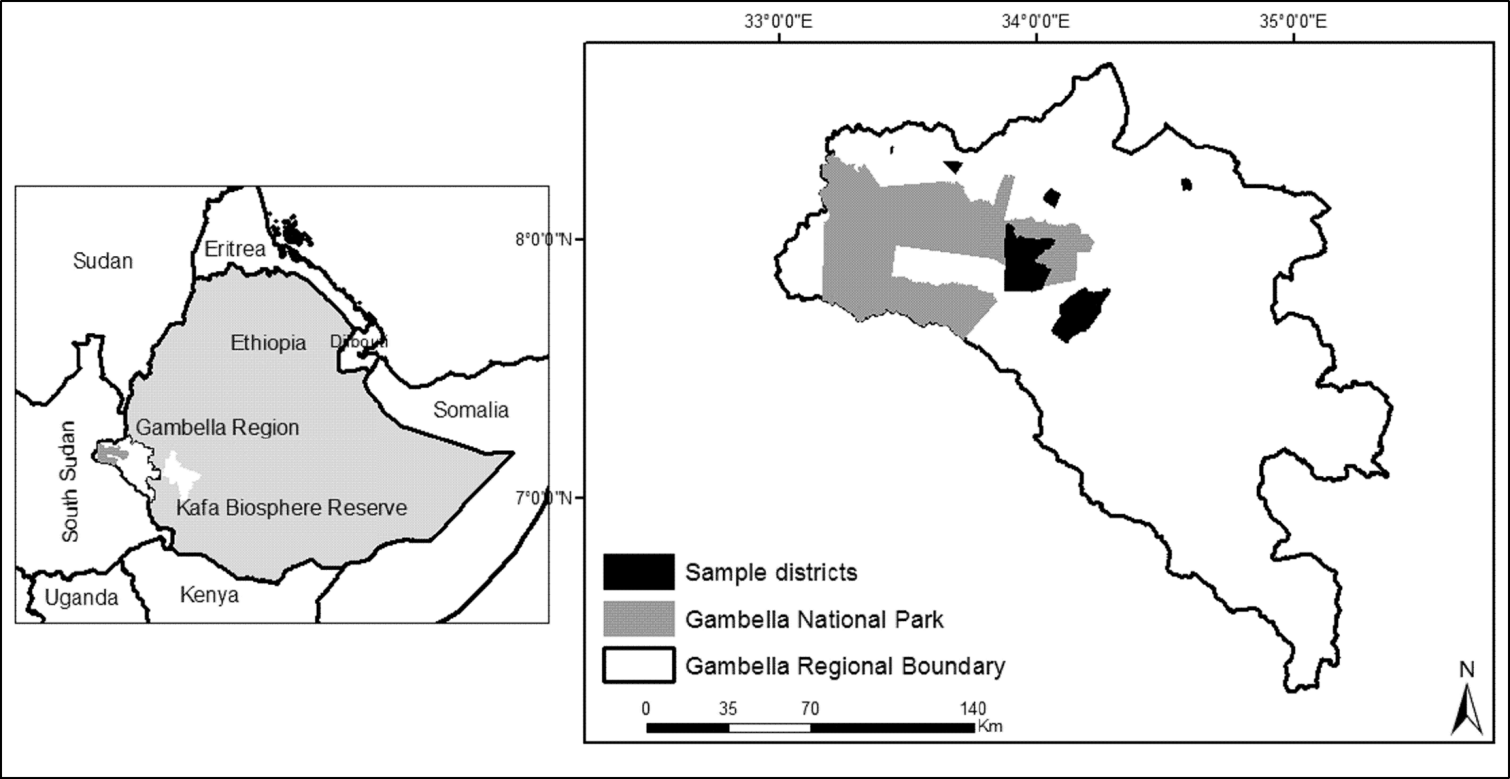
Map of study area.

GNP is located in Gambella, 850 km west of Ethiopia’s capital Addis Ababa (Fig 1). The region has a population of approximately 365,000, with two main ethnic groups, Anuak (21% of total population) and Nuer (46% of total population) [31]. The Anuak are resident agriculturalists, fisherfolk, and hunters, and the Nuer are pastoralists and agro-pastoralists [32, 33]. The Anuak villages are located along river banks, encompassing most of the 0.5% fertile alluvial riverine land in the region [34]. Hunting is also common and bushmeat is a major source of protein for locals [35, 36]. Resource-based conflicts are common between the Anuak and Nuer [32–34] and GNP can be preconceived of as a third party in the competition for resources.

GNP was established upon the recommendation by F. Duckworth, a British hunter and game warden who was contracted by the central/imperial government to assess the situation in the region in the 1970s [37]. Duckworth [37] suggested immediate action to protect the wildlife by establishing a National Park and a hunting moratorium for at least five years. Based on Duckworth’s report, in 1973 the Ethiopian Wildlife Conservation Organization decided to establish a reserve flanked by two hunting areas, assuming that it would be difficult to institute strict conservation reserves in an area without control and little reach of the state [37], but a year later revised its stand and established GNP. At the time of its establishment, GNP was the largest national park in Ethiopia, with an area of 5,061 km^2^ [37]. However, its size and borders were modified in 2011, following land transfers to investors for large scale mechanized commercial agriculture (‘land grabs’) [38]. Currently, the total area of GNP is 4,575 km^2^ and it now borders South Sudan. GNP is ‘soft edged’: boundaries are not visible and the transition to surrounding land use is gradual. GNP here refers to the National Park and its immediate zone of influence.

GNP is generally flat, with elevation ranging from 400 to 768 m above sea level [39]. The higher elevation areas have deciduous woodland and savanna, but the park’s most distinct feature is the floodplain located between Baro and Gilo rivers [35]. Due to its transboundary migratory ecosystem [35, 40], there is an initiative to integrate GNP into a transboundary protected area system with Boma National Park in South Sudan [41]. During the time of our data collection, the Ethiopian Wildlife and Conservation Authority was co-managing GNP with African Parks Network, a non-profit conservation organization specializing in long-term management of protected areas in partnership with the country’s government. However, African Parks Network terminated their contract with the Ethiopian government and left the country in 2016.

### Data collection and analysis

This study was approved by the Oklahoma State University Institutional Review Board (approval number AS 1434). Local research permits were acquired from Gambella regional and district level administration offices. A printed descriptive summary of the research was given (or read out) to all participants in key informant interviews, household surveys, and discussions, and consent was obtained orally from all participants. We interviewed key informants (i.e., government and NGO officials and experts) and household owners and conducted informal discussions with local people in and around GNP to assess their attitudes towards lions. We trained three park scouts from GNP as data collectors and conducted trial interviews in Gambella town to test the data collectors and to ensure that all questions were clear. Participation was voluntary since respondents were not paid.

Our data collection focused on: estimating the level of livestock depredation attributed to lions, assessing local knowledge about carnivores, evaluating tolerance and retaliatory actions towards lions, and documenting attitudes towards and cultural value of lions. Data were collected with two techniques: key informant interviews and household survey using questionnaires (S1 File). The household survey data were collected from May 2015 to December 2015 while key informant interviews were conducted from April 2015 to December 2016.

Six key informants with work experience in and around GNP were interviewed after obtaining individual verbal consent. The interviews were semi-structured and were conducted in informal settings. Questions focused on the status of lions, livestock depredation by lions, retaliatory killings, and challenges of conserving lions in Gambella. We also obtained accounts of reported lion attacks and lion killings in and around GNP from the headquarters of GNP.

The household surveys were conducted in the town of Gambella (the regional capital) and in five different districts: Wentwa, Puldiang, Ngenngang, Ulaw, and Puchala. The first three districts were from the Nuer zone, the part of Gambella where locals’ livelihood is based on pastoralism, more so than in the Ulaw and Puchala districts from the Anuak zone. For the survey, we randomly selected 35 household heads (respondents hereafter) in each district from the list of household head names provided by each district office. If a household head was not present at the time of interview, household heads one door to the right of the selected one were interviewed. The questionnaire required about one hour to complete.

The questionnaire had four sections. The first section assessed the demographic and economic status of respondents. The second section assessed respondents’ perception, attitude, knowledge of, and coping with lion population in and around GNP and was comprised of 15 questions using a Likert scale from 1 (strongly agree) to 5 (strongly disagree). The third section gathered information about respondents’ broad knowledge about carnivore species. In this section respondents were asked to identify carnivores and their tracks by looking at photos of six carnivores,(African wild dogs (*Lycaon pictus*), spotted hyena (*Crocuta crocuta*), jackal (*Canis mesomelas*), leopard (*Panthera pardus*), lion (*P*. *leo*), and serval (*Leptailurus serval*) and four carnivore tracks (African wild dogs, spotted hyena, leopard and lion). The last section of the questionnaire assessed the problem of lion attacks on humans and livestock, people’s preventive actions, reasons for lion attacks, and the trend of these attacks in the past five years. We also asked how much livestock is lost to disease and theft.

Additionally, we held opportunistic informal discussions with individuals or groups of people. There was no overlap between participants in these informal discussions and key informants or household survey subjects. These discussions occurred based on self-initiated conversations by locals about our activities in their community or by individuals approaching us with information that they thought might be of interest to us. During all informal discussions, we communicated to the participants that their responses might be reported anonymously and we obtained their verbal consent to proceed.

We converted the different types of livestock to Tropical Livestock Units [42] to obtain a standardized value that is comparable across stock types. We used descriptive statistics and selected Likert scale questions that measured attitude; to assess internal consistency of data we calculated Cronbach’s α. To investigate the variation in attitude we ran multivariate generalized linear mixed models (GLMM) [43] in R. We considered district name a random effect and the following eight predictor variables as fixed effects: gender, education level (illiterate, reading and writing, elementary school, high school, and college diploma and above), occupation (agriculture, government employee, pastoralism, and dependent), where people live (rural or town), total number of livestock, livestock loss due to lions, livestock loss due to theft, and livestock loss due to disease. The response variables used to measure attitude in three separate GLMM analyses were represented by the following three survey questions: ‘Do you want lion numbers to increase in Gambella?’, ‘Is it important to conserve lions?’, and ‘Do you want lions to be extirpated from your community?’.

To compare locals’ attitude data from inside and around GNP with those published for Kafa [10], we computed a composite attitude scale (or index) by calculating the mean of responses for identical Likert Scale questions that measured attitude. To do this, we assigned values to responses (5: strongly agree, 4: agree, 3: neutral, 2: disagree, 1: strongly disagree) and multiplied the count of respondents for each question with its assigned value, summed the values, and divided the sum by the total number of respondents. We also ran Mann Whitney U test, a non-parametric test that deals with ordinal variables with no definite distribution, [44], to measure differences in attitude between Kafa and Gambella.

## Results

A total of 210 respondents participated in the household survey (S1 Table); the majority of these respondents were males (N = 147; 70%) born in Gambella (N = 196; 93.33%), where they lived their whole lives. Half of the respondents (49.5%) were from the agricultural sector, 24.3% were government employees, 11.4% were dependent, 8.6% were self-employed, and 6.2% were employees of private companies and daily laborers. Among those whose occupation was agriculture 53.85% were pastoralists, 7.69% agro-pastoralists, and 38.46% practiced crop farming and animal husbandry. The majority of our respondents (70.5%) did not own land, and only 23.3% owned more than 0.5 ha. Almost all (96.67%) of our respondents disclosed their monthly income, which ranged from less than or equal to 500 Ethiopian Birr (approximately 22.22 USD; 16.67% of respondents) to over 3,000 Ethiopian Birr (approximately 133.33 USD; 23.33% of respondents). These income estimates do not include earnings from informal economies and do not indicate consumption levels.

### Knowledge about carnivores

Almost all respondents could identify lions (98.6%) and spotted hyenas (97.6%) from the given set of photos. Other carnivores that were recognized by a large percentage of respondents were leopard (87.6%), jackal (68.1%), and serval (67.6%). African wild dog was the least recognized carnivore (37.6%). Most respondents had seen spotted hyenas (87.6%) and lions (85.7%) at least once in their life, whereas only 17.6% had seen African wild dogs. Similarly, the majority of our respondents could identify pictures of lion (70.5%) and hyena (60%) tracks, while only 3.3% were able to identify tracks of an African wild dog. Furthermore, all key informants (100%) agreed that the lion population is declining in Gambella, although no actual data confirming this trend exist.

### Lion attacks and lion killings

**Fig 2:**
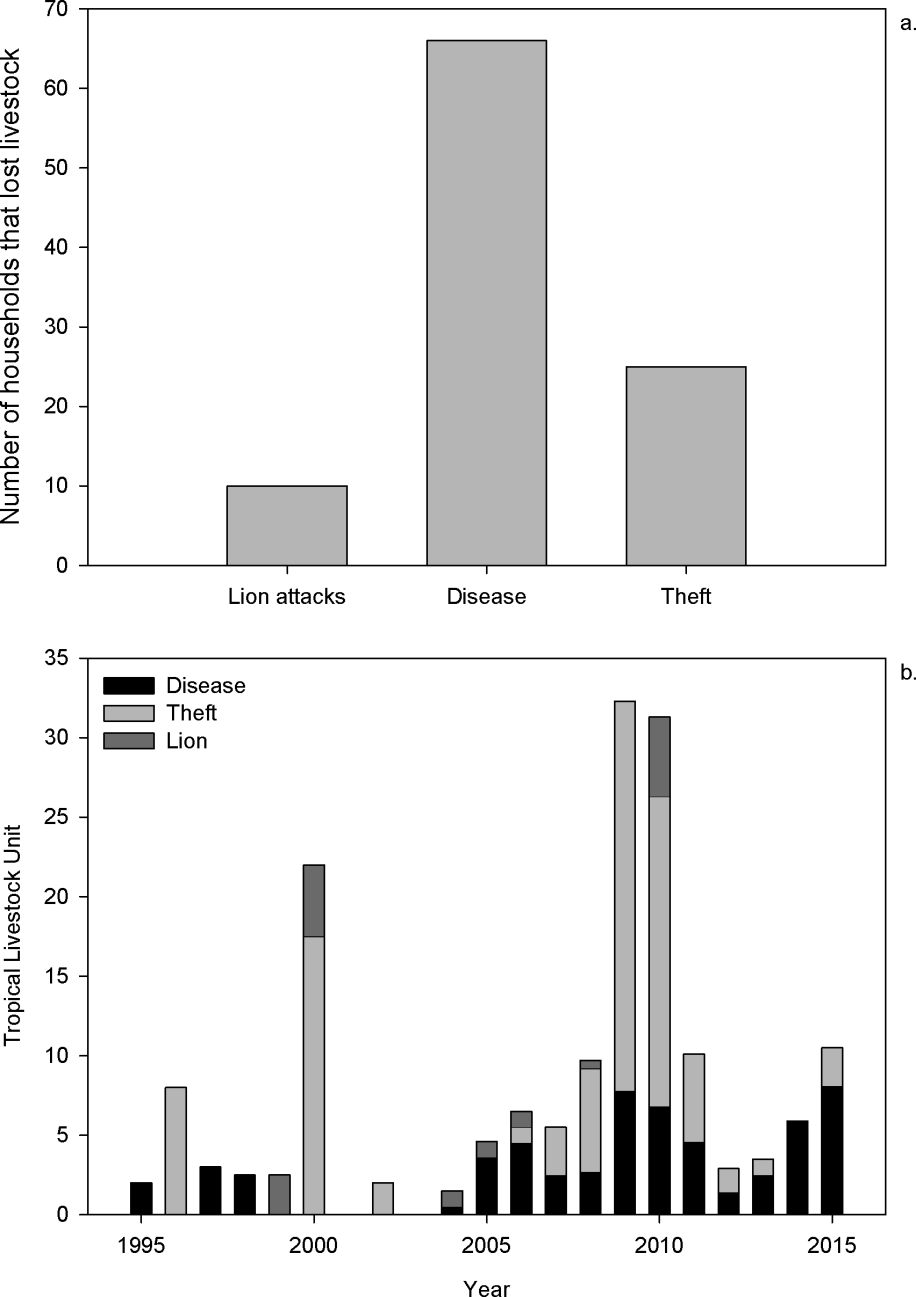
Overview of livestock loss between 1995 and 2015 in Gambella region, Ethiopia. Three main factors linked to livestock loss are presented in relation to (a) number of individuals experiencing livestock loss and (b) magnitude of livestock loss, in Tropical Livestock Units, by each factor; a total of 210 household surveys were analyzed.

Overall, our survey revealed three lion attacks on humans and 31 livestock depredation incidents on seven households. All three recorded human attacks happened before 2000, and the last depredation incident occurred in 2010. A single respondent reported almost a third of the depredations (10), all in 2010. Overall, the respondents identified diseases as the most frequent factor for livestock loss (Fig 2A), although theft (reported by respondents) caused the highest amount of livestock loss, as measured in Tropical Livestock Units (Fig 2B). However, none of our respondents mentioned theft or disease as issues in the last section of the questionnaire (comments and concerns regarding livestock) or during informal discussions.

All six key informants (100%) stated that livestock depredation by lions is a serious problem, and that this problem is more pronounced in the Nuer zone due to the higher number of livestock. According to the key informants, human-lion conflicts increase in the wet season (June to November), when the plains are flooded and most of white eared kob (*Kobus kob*), an important prey item for lions, migrate to South Sudan. During floods lions also shift closer to villages to escape the high waters and as a result depredation increases.

**Fig 3:**
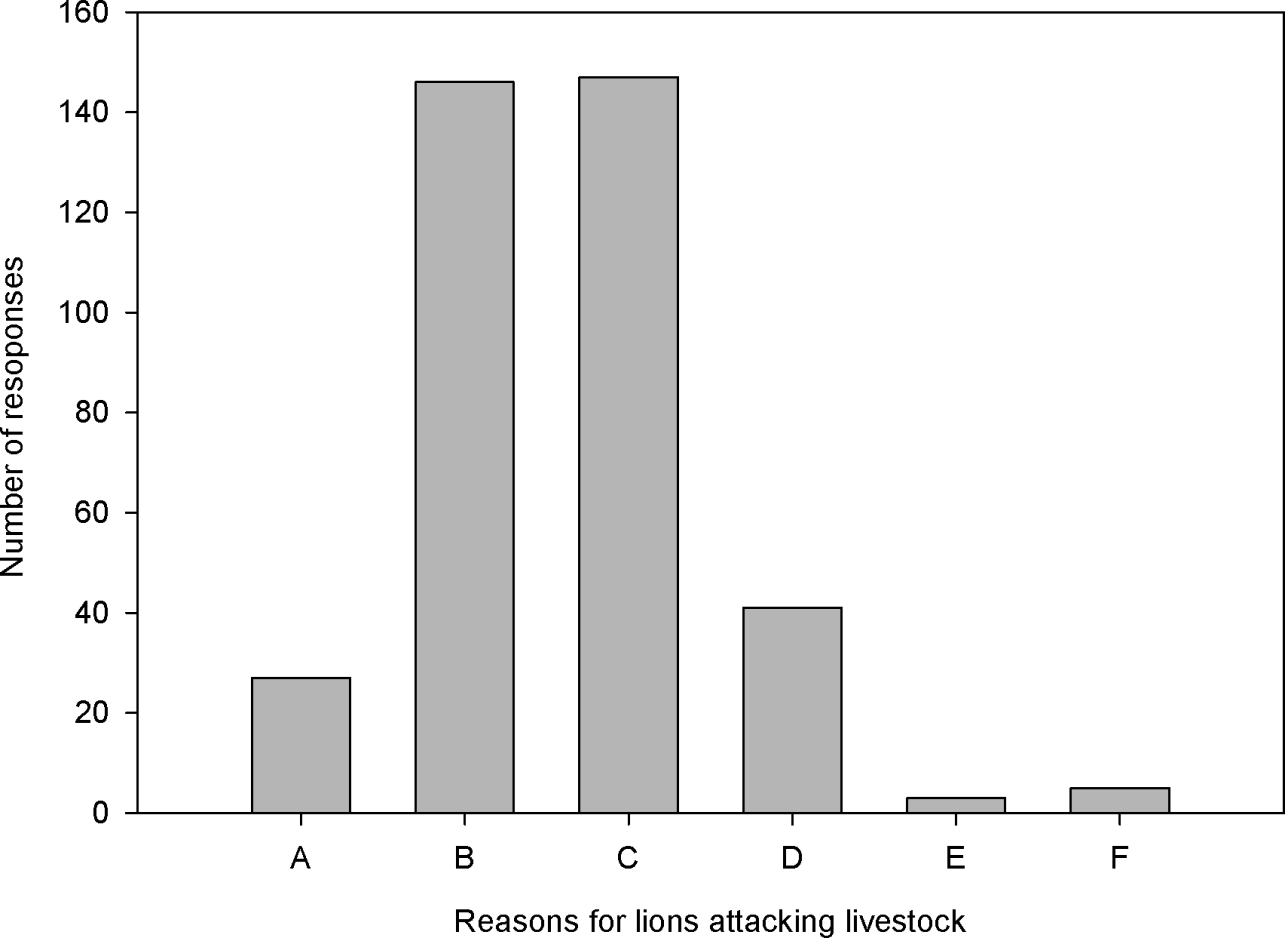
Locals’ answers to the question ‘why do lions attack livestock’ in Gambella, Ethiopia (A = lack of wild prey; B = because the livestock graze close to lions; C = because lions are violent in nature; D = because lions are habitual raiders; E = don’t know; F = other reasons) (N = 210).

Most of our respondents thought that depredations occur because livestock graze close to (and in) lion habitat and because lions are violent in nature (Fig 3). The majority (60.9%) of our respondents believed that depredation had decreased in the past five years, while 33.8% responded they did not know whether depredation had increased or decreased.

The majority of our respondents (90.9%) stated that lions are not hunted in Gambella. However, in informal discussions, at least three people from each village mentioned that any wildlife should be killed if it is a problem animal (if it attacks people or domestic animals, or if it damages crops). The few respondents (9.1%) that answered ‘lions are hunted in Gambella’ gave the following reasons: to protect livestock from attacks (84.2%), because lions are dangerous and should be kept away from the human environment (10.5%), and because cattle rustlers kill lions to use their skin to make the stolen cattle run faster (5.3%). The last reason came up often in informal discussions with respondents that said ‘lions are not hunted in Gambella’. The same group of respondents mentioned frequently during informal discussions that people living in and around Metar district kill lions for their skin because lion skin is used to make jewelry in Metar and in adjacent areas in South Sudan.

All key informant interviewees mentioned that hunting by the region’s Special Police is a problem that has been reported by GNP officials to the regional government to no avail (*Special Police are police forces that receive para-military training and are armed to protect key regional facilities*. *They will intervene in situations that cannot be controlled by the conventional police*, *as a last resort of the regional government*, *before it appeals to the federal government for intervention*). One key informant stated: “the Special Police seem to have a wrong notion that the general rules do not apply to them, so they kill wildlife as they please; and they carry the kills in the open too, unlike locals who try to hide when they see a GNP vehicle or staff”. On the other hand, all key informants (100%) stated that lion killing is a problem in Gambella, but since it is an illegal act, locals do not report killing incidents, unless park scouts or other GNP personnel find the carcass in the field. During one of our data collection seasons, we found one lion carcass.

Key informants mentioned that local people do not formally report depredation incidents as they happen, but raise the issue whenever they have the opportunity to meet with GNP staff formally or informally. As a result, depredations are reported intermittently to GNP staff (Table 1). Some of the key informants stated that local people are starting to demand compensations from GNP for their claims of livestock loss. In 2015, local police officers killed a lion, claiming that it attacked their car. Two of our six key informants mentioned that some South Sudanese refugees are armed, further exacerbating the problem of poaching.

**Table 1:** Livestock loss (expressed in Tropical Livestock Units, TLU) due to lion depredation reported by local population to staff of Gambella National Park, Ethiopia.

Place	TLU	Year
Gambella town	4	2003
Metar	6	2004
Lare and Jikao	10	2010
Lare and Jikao	10.5	2016

### Attitudes towards lions

A little more than half of our respondents (53.8%) do not want lion numbers to increase in Gambella. Among these respondents, more than half (62.83%) did not give a reason, 19.47% responded that it is because lions will kill people and livestock, 14.16% replied ‘it would just be bad’ if lion numbers increase, and 3.54% gave the reason that lions are useless.

According to most of our respondents (73%), lions do not have any cultural value in Gambella. Additionally, the majority (57.6%) think that lion presence is not advantageous or does not benefit humans or the environment. The majority (67.62%) of our respondents answered that they like seeing lions in the wild, but when asked if lion killing should be allowed by law about half (52.4%) of our respondents answered yes. Only about 17.62% of our respondents want to see lions extirpated from their community, while 63.33% believe that it is important to conserve lions. Most of our respondents (83.33%) prefer the lions confined within a restricted area, like GNP. Our index of internal reliability (Cronbach’s α) was 0.678, slightly lower than the ideal cutoff value of 0.7 [45]. This suggests that 67.8% of the variability in our attitude data represents the true score of what we measured.

The GLMMs revealed that education level and livestock loss due to theft significantly affected whether respondents wanted lion numbers to increase (coefficient estimates of 0.098 and 0.039, respectively; both P<0.05). Livestock loss due to lions and respondent’s gender had negative coefficient estimates that were almost significant (coefficients -0.13, P = 0.07 and -0.13, P = 0.06, respectively). Livestock loss due to lions significantly affected responses regarding the importance of conserving lions and wanting local lion extirpated (coefficient estimates of -0.29 and 0.39, respectively; both P<0.01). Total livestock number was also a significant factor in assessing if locals wanted lions to be extirpated from their communities (coefficient estimate = 0.04, P<0.05).

### Contrasting GNP with Kafa Biosphere Reserve

**Fig 4:**
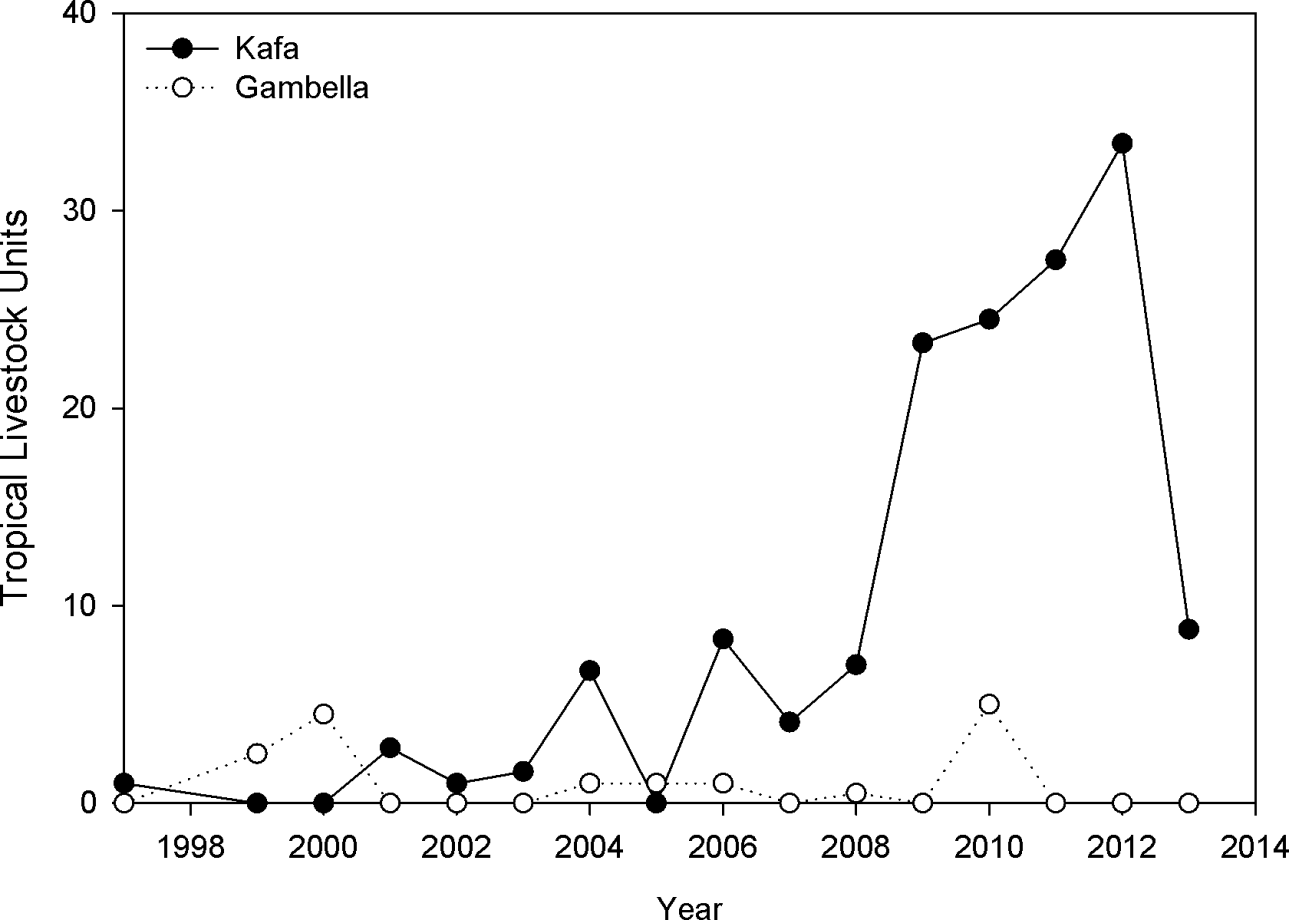
Comparison of livestock loss reported by individuals surveyed in Kafa Biosphere Reserve and in and around Gambella National Park, Ethiopia.

More economic loss is caused by lions in Kafa Biosphere Reserve (southwestern Ethiopia; hereafter ‘Kafa’), than in and around GNP (Fig 4) [10]. Between 1999 and 2013, communities around Kafa reported about 10 times higher loss in Tropical Livestock Units compared to communities in and around GNP.

The composite attitudinal indices differed between Kafa and GNP. In Kafa, the highest value was for the importance of conserving lions, while in and around GNP it was for concerns over depredation by lions. Results of the Mann Whitney U test show significant attitudinal differences between Kafa and GNP, except for wanting lions to be extirpated from their respective communities (Table 2).

**Table 2:** Mean survey responses to statements that measured attitude in Kafa Biosphere Reserve and in and around Gambella National Park in Ethiopia, and the p-value of Mann-Whitney U test for differences between the two areas.

Statements	Gambella	Kafa	p-value
I like seeing lions in the wild	3.88	4.17	<0.001
It is important to conserve lions in Gambella/Kafa	3.78	4.54	<0.001
Presence of lions is a sign of a healthy environment	3.52	4.08	<0.001
I want lions extirpated from my community in Gambella/Kafa	3.62	3.65	0.6774
Depredation by lions is a very concerning issue in Gambella/Kafa	4.16	3.88	0.01926

## Discussion

We found that disease and theft are the leading causes of livestock loss in and around GNP. Other studies of human-carnivore conflict also reported that large carnivores were incorrectly identified as the cause for livestock loss [46–48]. The last livestock depredation by lions in Gambella was reported from 2010, but the majority of our survey respondents believed that depredation increased over the past five years. Such mismatch between depredation and locals’ perception of risks associated with lions has been reported in other studies. For example, local communities in Waza, Cameroon [49] and in Ruaha, Tanzania [47] considered depredation the top livestock mortality factor, when in reality disease was more important in both cases. This subjective perception may be due to the resentment towards large and conflict-prone species [50]. Although most respondents of our survey stated they do not want lions to be extirpated from Gambella, anecdotal evidence suggests that lion killing exists and that the lion population has declined in and around GNP. Despite the low overall impact of lions on local communities in Gambella, there is a widely accepted notion that lions are dangerous animals with no cultural value.

People’s attitudes and behaviors towards lions could be shaped by actual attacks or by perceived risk [51]. This is the case in GNP, demonstrating that peoples’ perception of risk often stems from rare and tragic events rather than many small ones with a greater cumulative and/or historic effect [52]. Underlying factors, different from livestock depredation, are shaping locals’ attitude towards lions in Gambella. One factor can be the inherent, natural fear of lions [53] which is one of the key factors shown to shape people’s attitude towards large carnivores [51]. We found education level to significantly affect locals’ attitude towards lions, as survey respondents with higher education levels were more supportive of the idea of lion numbers increasing in Gambella, similar to the findings of Roskaft et al. [54] in Norway and Lagendijk and Gusset [9] in South Africa. This may be because higher education level has been linked to increased naturalistic scores (valuing outdoor recreational contact with wildlife) [55]. In our study, respondents’ decreasing support for the idea that it is important to conserve lions and increasing support for the idea that lions should be eradicated from the community were explained by amount of livestock loss to lion depredation. Thus, our study suggests that livestock depredation shapes locals’ attitude towards lions in Gambella. In addition, with increased number of livestock, the desire to see lions extirpated from the community also increased significantly in our study. This is an indication that human-carnivore conflicts shape opinions of conserving large carnivores due to the fear of attacks [56]. We also found a positive relationship between livestock loss to theft and the idea of increasing lion numbers in Gambella. One possible explanation for this conflicting result could be that survey participants consider economic benefits of livestock to any person to be a better situation than livestock loss to lions and disease, when no person receives economic benefits.

Human-carnivore conflicts foster negative attitudes, leading to unfavorable behaviors towards large carnivores. However, depending on local cultures, some communities may be more tolerant than others to presence of carnivores, as in Kafa, southwestern Ethiopia. Although lion attacks are more frequent in Kafa than in and around GNP, communities in Kafa have many cultural beliefs about lions that dissuade from lion killing, thus creating the platform for coexistence [10]. In contrast, most of our survey respondents stated that lions do not have a cultural value in Gambella, which indicates that local culture cannot be used to promote tolerance and coexistence like in Kafa. This underlines that issues of–and potential solutions to–human-lion conflicts should be understood and designed at a socially meaningful regional scale [30]. The opposite levels of intrinsic motivations in and around GNP and in Kafa towards tolerating lion attacks imply that approaches to these communities must be different when planning lion conservation actions [56]. Potential solutions for Gambella should also take into consideration inter-ethnic conflicts that are caused by border disputes and demands of political legitimacy [57].

Trophy hunting has a long history in Gambella, as it was one of the commercial activities that the region was known for [58]. Anecdotal evidence and informal communication with key informants show that lions are persecuted in Gambella. Additionally, the region’s human population has increased due to relocation of Ethiopian highland settlers [34] and South Sudanese refugees, increasing the need for resources and likely leading to more poaching and land clearing. Poaching can affect lions directly, by decreasing lion population, or indirectly, by reducing prey availability. Thus, law enforcement should be a priority to reduce poaching, as increasing law enforcement has been linked to reduction in illegal hunting activities [59, 60]. We strongly recommend including all Gambella stakeholders (local people, refugees, and the Special Police) in conservation actions. Additionally, the national level authority (Ethiopian Wildlife and Conservation Authority) needs to engage in discussions regarding the mandate of GNP staff to enforce the law, however weak and regardless of who is breaking the law.

Law enforcement is the retribution aspect of the carrot-and-stick approach in managing conflicts. The reward aspect of this approach takes two forms: increasing tolerance of local people to presence of carnivores and reducing conflicts [61]. These could be achieved through activities like supporting local schools, providing conservation education and economic incentives, and removing problem animals [62]. The conservation education should include two components: instrumental (predetermined guidelines) and emancipatory (local people part of decision making) [63]. Conservation education has been shown to create a positive perception towards carnivores [64, 65]. In the case of GNP, communicating to locals that disease and theft are the major causes of livestock loss could reduce the misdirected resentment towards lions and foster tolerance. Although the economic consequence of livestock loss due to depredation is overall smaller than loss due to disease and theft, it has a significant conservation implication. This is particularly important because perceived carnivore harm has been found to be more influential than actual attacks in guiding coexistence with carnivores [13]. Since some of our key informants stated that depredation increases in the wet season in Gambella, strict practices of mechanisms that limit livestock depredation [66] in the wet season could be part of the community conservation education. All of the above fall under the instrumental education aspect. The emancipatory approach could include locals taking part in discussions that guide conservation policies and managing conflicts. Interventions through education and outreach programs have been shown to have weak impacts on altering behavior [17, 67]. Hence, we suggest coupling the conservation education with “the stick”: strict law enforcement for bringing change in behavior towards lions in Gambella [17, 68].

**Fig 5:**
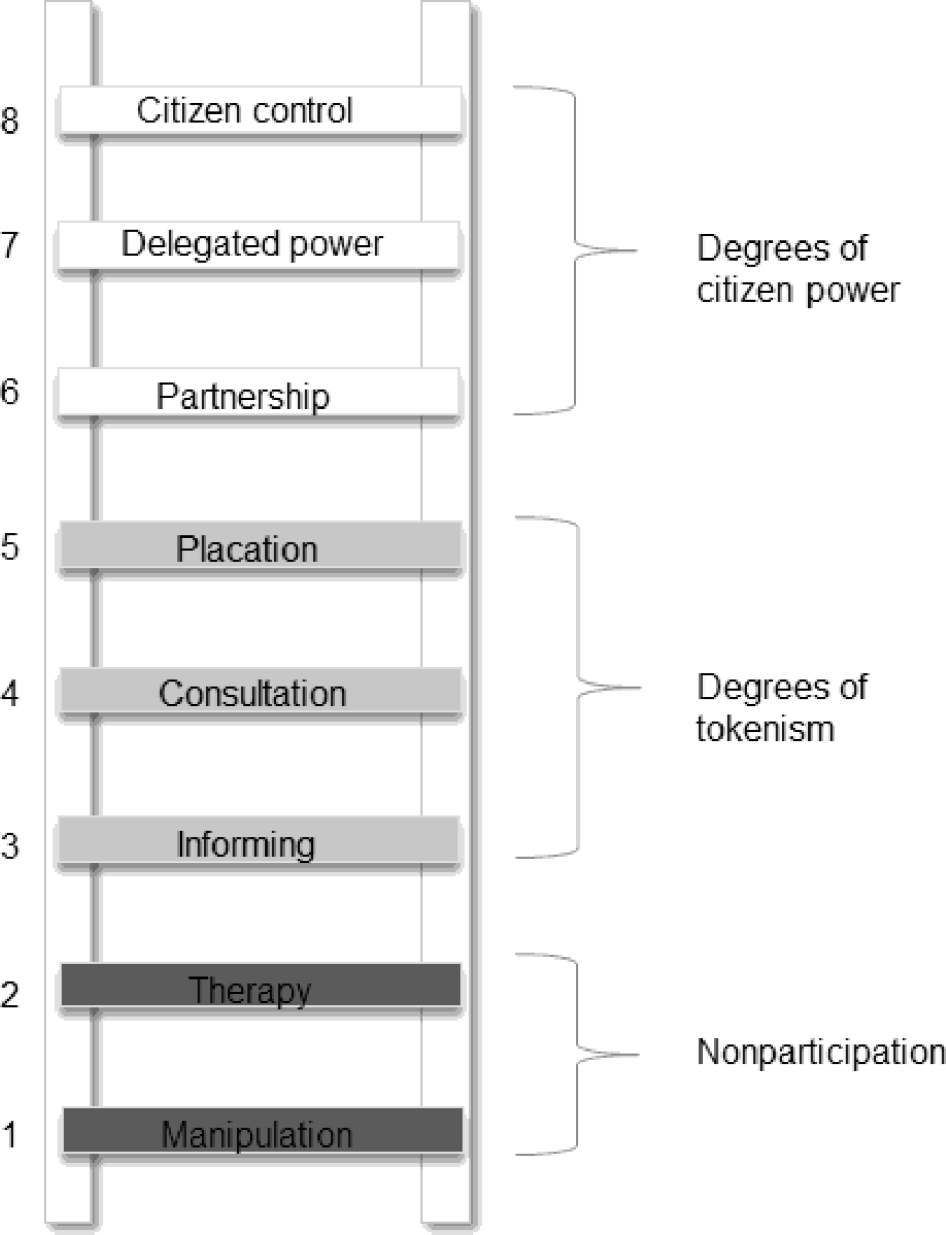
The eight rungs of Arnstein’s ladder of participation, grouped into increasing degrees of power (source: [16]).

Bottom-up conservation is challenging in places with little to no community engagement in conservation and where participants have no decision making power or where power is not balanced between participants [69]. However, Moeliono [70] suggests that in places where conservation is the responsibility of governments, implementing local participation can be guided by steps in Arnstein’s ladder of participation [18] (Fig 5). The carrot-and-stick approach mentioned above encompasses the bottom five rungs of the ladder grouped into two levels: the non-participation (local people with no actual decision making power) and the degrees of tokenism (local people as part of the dialogue but not making decisions) [18]. After empowering local people with skills and capacities, communities can be guided up the ladder for true participation, represented by the top three rungs: partnership, delegated power, and citizen control. The success of this true participation will depend on individuals or groups selected and making the process relevant to local needs and priorities [69].

Land use complicates conservation actions. The allocation of large tracts of land for commercial farming has intensified in Gambella, with about 545,178 ha of land (16% of the region’s total area) leased between 2003 and 2014 [38]. The federal authority leasing land did not use the GNP boundaries established by Ethiopian Wildlife and Conservation Authority and as a result, has leased GNP land [71]. This was partially solved with the re-demarcation process of the park in 2011, but some agro-investments still exist inside GNP, some not yet in use. GNP is not gazetted, and with more land-leasing, another re-demarcation might occur. Our data suggest no impact by these farms on the status of human-lion conflicts. However, the pressure for land can affect the lion population by causing prey depletion and habitat fragmentation. It may also increase the human-lion interface, which could lead to more conflicts. The impact of leasing “unused but protected” land [71] on lion populations (and other wildlife) needs to be studied.

A positive attitude towards the presence of carnivores, mostly attributed to urbanites, might be considered as a way of dominance over rural residents [30]. Additionally, national parks created with disregard for local interests face challenges in serving their purpose. This is because parks mostly overlook the political agency of local communities [72], considering locals only as resource users, and potentially leading to everyday forms of resistance [73]. This compels us to question if lion killing in the Gambella region is an enactment of a ‘natural right’ or if it is a way of resistance to a larger issue, an unfavorable protected area (GNP). Future research should investigate if changes to lifestyle and livelihood of local people are causing resistance to conservation efforts [74]. Research should clarify whether local people resent the idea of conservation because it affects their lifestyle and livelihood and perceive killing wildlife as a way of pushing back [75, 76]. GNP is considered to support a viable lion population in Ethiopia [26], but without interventions to understand and address the threats, this could change.

## Supporting information

S1 File Questionnaire used for household survey data collection. (PDF)

S1 Table De-identified household survey dataset with keys for coded responses. (XLSX)
