# Supplementary material for: Beyond the numbers: Human attitudes and conflict with lions (Panthera leo) in and around Gambella National Park, Ethiopia
Source: PLoS One. 2018 Sep 25;13(9):e0204320. doi: 10.1371/journal.pone.0204320 (PMC6155518; doi:10.1371/journal.pone.0204320)
Supplement: S1 File — (PDF) [file pone.0204320.s001.pdf]

Date: _______________ Coordinates of the house/location: __________________________

I. Socio-economic characteristics of the respondent

1. District: _________________________
2. Sex: ___________
3. Date of birth: _________________________
4. Place of birth: _________________________
5. How long have you lived in Gambella? _________________________
6. Education level: A. Illiterate B. Read and write C. Primary/middle school D. High school E. Diploma and above
7. What is the family composition of your household?
   Spouse ________ Children ____ (M) _____ (F) Relatives______ Others_________
8. Occupation

II. Economic level of the household

9. Average land holding in hectares?
   a. <0.25 b. 0.26-0.5 c. 0.51-0.75 d. 0.76-1 e. >1 f. I don't have a land
   (If you said you do not have land, please pass to question 13)
10. Do you produce crops? a. Yes b. No
11. If yes, is your harvest usually enough to feed your family? a. Yes b. No
12. How long do you generally consume your harvest before you start buying food?
13. What is the estimated average total household monthly income in ETB?
    a. ≤500 b. 501-1000 c. 1001-2000 d. 2001-3000 d. >3000

III. Lion management, knowledge and perception

14. Pleas tick the alternative that the best describes your opinion
    (Key: 1-Strongly agree, 2-Agree, 3- Neutral, 4- Disagree and 5- Strongly disagree)

No.	Questions	1	2	3	4	5
1	Lion is bad animal					
2	The presence of lions is a sign of a healthy environment					
3	Depredation by lions is a very concerning issue in Gambella					
4	Lions are known for attacking and injuring people					
5	I would be afraid to go into the forest/filed if there are lions					
6	Lion is dangerous to humans					
7	It is important to conserve lions in Gambella					
8	I like seeing lions in the wild					

9	I want lions extirpated from Gambella					
10	Lions should only live in restricted places in Gambella					
11	Killing of lions should be strictly regulated by law					
12	Killing of lions should be allowed by law					
13	Lions have ample prey in the wild					
14	The number of lions in Gambella has notably increased in the past ten years					
15	Lions habitat destruction is a problem in Gambella					

IV. Carnivore knowledge

15. Which carnivore species do you recognize from the pictures?
1. ________ 2. ________ 3. ________ 4. ________ 5. ________ 6. ________

16. Which species (from the pictures in Qn #15) have you ever seen?
1. ________ 2. ________ 3. ________ 4. ________ 5. ________ 6. ________

17. Which of the tracks can you identify (from the provided picture of tracks)?
1. ________ 2. ________ 3. ________ 4. ________ 5. ________ 6. ________

18. According to you, among the above given carnivores (in Qn #15) which are the most dangerous? (Give a score: 1: extremely dangerous, 2: very dangerous and 3: dangerous)
1. ___________________ 2. __________________ 3. _____________________

Why do you think these carnivores are dangerous?
____

19. Do you want lion numbers to increase in Gambella? a. Yes b. No
Why? ____

20. Do you think lions have any advantages? a. Yes b. No
If yes, please mention some of their benefits?
____

21. Do lions have a special meaning/importance in your culture? a. Yes b. No
If yes, please explain in detail:
____

22. Do you know any carnivore body parts that are used for preparing traditional medicines?
a. Yes b. No; If yes, please explain in detail:
Which animal? _________________

Which part of its body? ___________________________
For which disease? ______________________________________
23. What are the common prey types for lions in Gambella?
____

24. Do people kill lions in Gambella? a. Yes b. No
If yes, please explain why
____

V. Livestock depredation

25. Do you have livestock? a. Yes b. No
If yes, please fill the table below. If No, please move to question 33.

Livestock species	Sex		Age level		
	Male	Female	Young	Adult	Old
Cow					
Donkey					
Sheep					
Goat					
Ox					
Others					

26. Did you lose livestock as a result of lion depredation? If yes, please fill the table below.

Species	Sex	Age	Number	Depredation place	Year
Cattle					
Donkey					
Sheep					
Goat					
Others					

27. Do you think you can avoid depredation?   a. Yes   b. No

28. What method you use to limit/avoid livestock depredation?
    a. Dog   c. Guard/Shepherd
    b. Enclosures/Fences   d. Fire
    e. Others; please specify: ____________________________________

29. According to you, among the above given depredation mitigation options, which are effective? Give a score of 1 to 3: from the most effective to effective)
    1.   2.   3.

30. How far away is your livestock grazing area from your house?
    Dry season _____________________Wet season _____________________

31. Have you ever lost livestock due to disease?   a. Yes   b. No
    If yes, please give details:
    a. Which animals did you lose? How many? ____________ ___________
    b. Which disease? ___________________________________
    c. When? Month __________________ Year __________________

32. Have you ever lost livestock due to theft?   a. Yes   b. No
    If yes, please give details:
    a. Which animals did you lose? How many? ______________ _____________
    b. Where were they stolen from and how? ______________________________
    c. When were they stolen? Month ______________ Year ________________

33. What can be a suitable remedial measure to reduce depredation by lions?
    a. Killing all lions
    b. Killing the problem causing individual lions
    c. Relocating all the lions
    d. Keeping livestock in a strongly fenced area
    e. Better protection of livestock
    f. Others; please explain ____

34. In your opinion; what is the trend of livestock attacks by lions these last five years?
    a. It has increased   c. It has not changed
    b. It has decreased   d. I do not know

35. Do you think people who lost livestock to lion attacks should be compensated?
    a. Yes   b. No
    Why? ____

36. When do you think the livestock predation by lions takes place?
    a. Mornings (6:00-12:00)
    b. Afternoons (12:00-18:00)
    c. Nights (18:00-23:00)
    d. Around and past midnight to Dawn (23:00-6:00)
    e. I do not know

37. In your opinion, why do lions attack livestock?
    a. Lack of wild prey
    b. Because livestock graze close to (and inside) lion habitats
    c. Because they are violent in nature
    d. Because they are habitual raiders
    e. I do not know
    f. Other reasons; please explain: __________________________________

38. Do lions attack people?   a. Yes   b. No

39. If yes, what preventive techniques do you use to avoid being attacked by a lion?
    ____
    ____

40. Has anyone from your immediate family been attacked by a lion?
    a. Yes   b. No
    If yes:
    a. What type of attack was it? ______________________________________
    b. Where did it happen? ____
    c. How did it happen? ____
    d. When did it happen?   Month ____________   Year ______________

41. Do you have any comments, observations or recommendations about livestock production, lion conservation, and the problem of depredation?
    ____
    ____
    ____
    ____
    ____
